# Dimensions within 24 weight history indices and their association with inpatient treatment outcome in adults with anorexia nervosa: analysis of routine data

**DOI:** 10.1186/s40337-019-0249-z

**Published:** 2019-06-10

**Authors:** Johannes Baltasar Hessler, Sandra Schlegl, Martin Greetfeld, Ulrich Voderholzer

**Affiliations:** 1 Schoen Clinic Roseneck, Am Roseneck 6, D-83209 Prien am Chiemsee, Germany; 2 Department of Psychiatry and Psychotherapy, University Hospital of Munich (LMU), Munich, Germany; 3 Department of Psychiatry and Psychotherapy, University Hospital of Freiburg, Freiburg, Germany

**Keywords:** Anorexia nervosa, Weight, Body mass index, Weight suppression, Set-point, Inpatient

## Abstract

**Objective:**

Next to weight suppression (WS), there are a range of less often examined weight history indices, and improvements to the WS construct have been proposed. We aimed to examine redundancy and overlap between 24 weight history indices in order to identify suitable constructs for further investigation.

**Method:**

Analysis of routine data of 770 female adult inpatients treated for AN. Twenty-four indices based on highest, lowest, and current weight, as well as developmental aspects were calculated and employed in correlational and factor analyses. The indices’ ability to predict core outcomes of inpatient treatment was investigated with regression analyses.

**Results:**

Five factors emerged: “WS and highest weight”, “weight elevation (i.e., difference between current and lowest weight since puberty)”, “lowest weight”, “age at past highest or lowest weight”, and “years since past highest or lowest weight”. The constructs within these factors showed high correlations. Most indices related to change in weight, ED psychopathology, as well as behavioral aspect of AN. While measures of WE related more to weight gain and general ED Psychopathology, indices including lowest weight were stronger predictors of changes in slimness ideal and inappropriate compensatory behaviors.

**Conclusion:**

Many proposed weight history indices are closely related and the amount of additional information in complex indices appears questionable. While highest weight seems to dominate indices of WS, WE may rely on current weight. These findings highlight that different aspects of weight history may relate to different aspects of current ED symptoms and their amenability to change under specialized treatment.

## Background

The physiological and psychological core symptoms of anorexia nervosa (AN) revolve around weight and low weight is the cardinal diagnostic criterion. The weight history of persons with AN or other eating disorders (EDs), however, only gradually moved into the focus of scientific attention. Most prominently, the concept of weight suppression (WS), which describes the difference between highest adult lifetime weight and current weight, was first examined in bulimia nervosa (BN) and later also in AN [1–3]. The more pronounced WS, the stronger the forces that pull weight back to its previous highest level [2], which is reflected in more rapid weight gain in persons with higher WS in BN [4] and AN [5–8]. WS is also associated with psychological facets of disordered eating, for example, increased bulimic symptoms [9].

In their conceptual review, Lowe and colleagues conclude that the constituents of WS are understudied and that the operationalization as applied above may not be fully adequate, as it does not relate the extent of WS to the highest weight or the expected weight for a certain demographic group [2]. As a consequence, they suggest that more detailed analyses of weight history should be conducted, including, for example, the age at the highest lifetime weight. Gorrell and colleagues pointed to a variability in calculating WS, which might account for some ambiguous findings [3]. For example, when calculating WS with weight only, two persons of different height and weight might show similar values. Hence, body mass index (BMI: kg/m^2^) suppression should also be examined. Two years earlier, though hardly considered in the literature, Schaumberg and colleagues proposed several alternative formulas for WS, which have not been further examined in longitudinal studies [10]. These authors first examined residuals from regressing current weight on highest weight as a measure of WS that is independent of the level of highest weight. These residuals were not associated with ED pathology in their sample, which lead to the conclusion that WS did not carry additional information compared to highest weight alone. Studies employing the standard operationalization of WS paint a mixed picture for the association between WS and highest weight (see Lowe and colleagues for an overview [2]).

Also surprising is the negligence of lowest adult weight in the literature. Only one study explicitly examined weight elevation (WE), which is described as the difference between current weight and lowest adult weight [11]. The authors introduced WE as a useful construct in aging populations that tend to gain weight over the years and whose WS might, therefore, equal 0. In their study with a community-based sample of older women, WE was associated with dieting, skipping meals, and extreme lifetime restriction. In general, higher values of WE might indicate a deviation from a persons desired low weight and, therefore, relate to body dissatisfaction [11], which in turn is associated with ED symptoms [12].

A further important, though seldom considered, problem arises from calculating weight history indices in young adults with EDs, who actually do not have a long weight history [3]. Contemplate, for example, the case of a woman of 19 years with AN, who reached her highest and lowest weights at ages 14 and 17, respectively. The simple calculation of difference scores for WS and WE does not capture that she probably never reached her normal adult weight. Hence, Lowe and colleagues suggested that BMI percentages, i.e., BMI at the age at which a person reached their highest or lowest weight divided by the 50th BMI percentile in the respective age and gender group, should be employed instead of highest or lowest past and current weight [1]. Resulting indices like BMI percentage suppression and elevation would be expected to incorporate developmental aspects of weight and ameliorate the shortcomings of weight history indices in young patients.

So far, it has not been examined whether the proposed adjustments to the indices and consideration of age at past weight actually translate to improved representations of weight history. Also, many indices would be expected to correlate highly (e.g. WS and BMI suppression), as they are transformations of each other. Therefore, the present study aimed to (1) detect redundancy and shared underlying constructs between the indices as well as (2) investigate their ability to predict weight gain and symptom change during inpatient treatment.

## Method

### Participants

Participants were female inpatients treated for AN between November 2005 and November 2012 in the Schoen Clinic Roseneck in Prien, Germany, which is highly specialized in the treatment of EDs. Inclusion criteria were a diagnosis of AN according to ICD-10 (F50.00, F50.01) including intense fear of gaining weight, a distorted body image, and a BMI lower than or equal to 17.5. Patients were diagnosed by experienced clinicians (all with a minimum master’s degree in medicine or psychology) during a standard intake interview. All patients gave written informed consent for scientific use of the routine data obtained during the inpatient treatment.

### Treatment

Patients received intensive multimodal treatment comprising individual psychotherapy (1 or 2 sessions of 50 min per week) and a disorder-specific manualized group therapy, both based on cognitive behavioral therapy. Individual therapy was not manualized, however, certain disorder-specific issues were addressed according to diagnosis-specific treatment programs, including psycho-education about eating disorders, individual case formulation, dietary changes, body exposure, body acceptance, cognitive restructuring, and relapse prevention. Other groups included art therapy, sports therapy, cooking training, and social skills training. Patients were obliged to eat normal portions three times per day and received mealtime support, however, were in charge of their eating and compensatory behavior. At all times, they had free access to a cafeteria, supermarket, and lavatories.

### Materials and procedure

At both admission and discharge, patients completed the Structured Inventory for Anorexic and Bulimic Disorders for DSM-IV and ICD-10 – Self-Rating (SIAB-S; Fichter & Quadflieg, [13, 14]). SIAB-S scores were employed to validate the diagnoses of AN and restrictive (AN-R) versus binge/purge (AN-BP) type by the clinicians.

Comprising seven subscales (bulimic symptoms, general psychopathology, slimness ideal, sexuality and social integration, body image, inappropriate compensatory behaviors, atypical binges) and a total mean score, the SIAB-S was shown to correspond well with the expert-rating version of the SIAB and constitute a reliable and valid tool for assessing ED symptoms [13]. Mean scores range between 0 and 4 and higher scores indicate more pronounced symptomatology. Further, the SIAB-S asks for highest and lowest weight since puberty, as well as age and height at the respective times. These data were employed to calculate a range of weight history indices. Current weight and height were measured at both admission and discharge and used to calculate pre- and post-treatment BMI.

### Weight history indices

We calculated a total of 24 weight indices, considering those proposed in the literature [1, 10, 11] and new ones, including BMI range and age-factored weight (AFW). Importantly, we did not exclude patients from the analyses, when their highest or lowest weights lay in their childhood or adolescence. Rather, we decided to calculate additional variables that included developmental aspects, e.g., BMI percentage suppression and AFW. Importantly, highest and lowest past weight are to be understood as “after the onset of puberty”. The following indices were calculated:

1. Highest weight in kg.
2. Lowest weight in kg
3. Highest BMI.
4. Lowest BMI.
5. Highest BMI percentage [1]:
  $$ \frac{highest\  BMI}{gender\ specific\ 50 th\  percentile\  BMI\  at\  age\  at\  highest\  BMI\kern0.5em } $$
  The values for the age- and gender-specific 50th percentile BMI here and, where applicable, in the following indices, were extracted from large German studies [15, 16]. Kromeyer-Hauschild and colleagues [15] reported BMI percentiles in age-intervals of half a year (e.g., for 18.0 and 18.5 years). We employed the mean of the respective pairs in our formulas. Lowe and colleagues suggested that for persons, who reached their highest weight at 20 years or older, the 50th percentile of age 20 should be used in the divisor, as physical maturation was completed at that age and any further weight gained was due to increased body-fat instead of developmental processes. Hence, indices for all patients aged 20 or older at the past weight were calculated with the 50th BMI percentile at age 20 in the divisor. The index describes the highest weight relative to what on average would be expected at a specific age and tackles the problem of weight history in adolescent or young adult patients.
6. Lowest BMI percentage (similar to [1]: Based on the same rationale as highest BMI percentage.
  $$ \frac{lowest\  BMI}{gender\ specific\ 50 th\  percentile\  BMI\  at\  age\  at\  lowest\  BMI\kern0.5em } $$
7. WS:
  $$ highest\ weight\ (kg)- admission\ weight\ (kg) $$
8. WE:
  $$ admission\ weight\ (kg)- lowest\ weight\ (kg) $$
9. BMI suppression:
  $$ highest\  BMI- admission\  BMI $$
10. BMI elevation:
  $$ admission\  BMI- lowest\  BMI $$
11. BMI suppression percentage [1]:
  $$ \frac{highest\  BMI}{gender\ specific\ 50 th\  percentile\  BMI\  at\  age\  at\  highest\  BMI\kern0.5em }-\frac{admission\  BMI}{gender\ specific\ 50 th\  percentile\  BMI\  at\  admission\  age} $$
  The index represents a measure of WS that was adjusted for developmental processes. As described above, for patients aged 20 or older at admission, the 50th percentile BMI for age 20 was entered in the divisor.
12. BMI elevation percentage (similar to [1]:
  $$ \frac{\  admission\  BMI}{gender\ specific\ 50 th\  percentile\  BMI\  at\  admission\kern0.75em }-\frac{lowest\  BMI}{gender\ specific\ 50 th\  percentile\  BMI\  at\  age\  of\ lowest\  BMI} $$
  The index represents a measure of WE that was adjusted for developmental processes.
13. BMI range:
  $$ highest\  BMI- lowest\  BMI $$
  The index describes the tendency of the organism to fluctuate between extreme weights and represents the ability to reach both high and low weights.
14. Percentage weight change (PWC; Schaumberg et al., [10]):
  $$ \frac{WS}{highest\ weight\ (kg)\kern0.5em } $$
  The index describes the degree of WS in relation to the highest weight and takes into account that a weight reduction of a given extent might have different implications for persons of different heights and, therefore, different highest weights.
15. Residual 1: residuals from the regression of admission weight on highest weight [10]. The residuals are orthogonal to highest weight and describe the variance in admission weight unexplained by highest weight. The orthogonality is of particular interest in WS, when the change score and its meaning depend on the initial value (i.e., highest weight). That is, high WS might result from extreme highest weight and/or low current weight and similar values of WS may actually have different meanings. The regression residuals solve this problem by indicating the amount of change independently from the regressor. That is, the index only contains information about WS that is not explained by the highest weight. Thereby, the index can be seen as a filtered version of other WS measures. Our sample yielded the following regression equation:
  $$ admission\ weight\ (kg)=0.179\times highest\ weight\ (kg)+ residual $$
16. Residual 2: residuals from regression of highest weight on admission weight [10]. The residuals are orthogonal to admission weight and describe the variance in highest weight unexplained by admission weight. Our sample yielded the following regression equation:
  $$ highest\ weight\ (kg)=0.615\times admission\ weight\ (kg)+ residual $$
17. Residual 3: residuals from regression of admission weight on lowest weight (similar to Schaumberg et al., [10]). The residuals are orthogonal to lowest weight and describe the variance in admission weight unexplained by lowest weight. Our sample yielded the following regression equation:
  $$ admission\ weight\ (kg)=0.741\times lowest\ weight\ (kg)+ residual $$
18. Residual 4: residuals from regression of lowest weight on admission weight (similar to Schaumberg et al., [10]). The residuals are orthogonal to admission weight and describe the variance in lowest weight unexplained by the admission weight. Our sample yielded the following regression equation:
  $$ lowest\ weight\ (kg)=0.799\times admission\ weight\ (kg)+ residual $$
19. Highest age-factored weight (h-AFW):
  $$ highest\ weight\ (kg)\times age\  factor $$
  This index was calculated as a measure of past weight that accounts for developmental aspects by factoring that weight with the age at which it was reached. For this purpose, we subtracted the age at which the highest weight was reached from 20 in order to gain a measure of temporal distance from completed physical maturation. The resulting difference was then transformed into an age factor, with negative values and 0 corresponding to a factor of 1, 1 to 1.1, 2 to 1.2, 3 to 1.3, and so forth. The index, hence, “punishes” an earlier age at which the highest weight was had, as normal development would lead to the highest weight at age 20. Also, the index “punishes” juvenile obesity, which is a known risk factor for ED pathology in adulthood [17]. Further, h-AFW is thought to be a simpler alternative to BMI percentage that could be used in clinical settings, where more complex calculations based on age- and gender-specific groups means (e.g., standardized BMI, PWC, or BMI percentage) are not available.
20. Lowest age-factored weight (l-AFW):
  $$ highest\ weight\ (kg)\times age\  factor $$
  Age at lowest weight was subtracted from 20 and the difference was transformed into a factor, with positive values and 0 corresponding to 1, − 1 to 1.1, − 2 to 1.2, − 3 to 1.3, and so forth. The index was calculated inversely to h-AFW in order to “punish” higher age at lowest weight, which suggests an inversion of normal development, as lowest age should occur before age 20.
21. Age at highest weight in years
22. Age at lowest weight in years
23. Years since highest weight: This index represents a measure of temporal distance corresponding to WS.
  $$ current\  age\ (years)- age\  at\  highest\ weight\ (years) $$
24. Years since lowest weight: This index represents a measure of temporal distance corresponding to WE.
  $$ current\  age\ (years)- age\  at\  lowest\ weight\ (years) $$

## Statistical analyses

The first aim of the study first was to detect redundancy among the indices and their components and map which indices measure similar constructs [2]. First, we calculated bivariate Pearson’s *r* regression coefficients for all indices. High absolute values would indicate strong redundancy between the constructs. Following conventional interpretation, we considered values of *r ≥* 0.70 as indicating large redundancy. Second, we conducted a factor analysis with a principal components method and varimax rotation of all indices in order to identify similar construct underlying the indices.

The second aim of the study was to investigate the ability of the indices to predict improvement during inpatient treatment. To this end, a series of multivariate regression analyses was calculated to assess the influence of the weight history indices on pre- to post-treatment change in BMI, as measured during physical examinations, as well as general ED psychopathology (SIAB-S total score), bulimic symptoms, slimness ideal, and inappropriate compensatory behaviors as measured with the SIAB-S (the other scales were excluded in order to reduce the number of analyses). Outcome variables were differences scores with higher values representing better improvement. The regression models for change in BMI were adjusted for length of stay (LOS) and all other models for LOS and admission BMI. The results include standardized regression coefficients, which allow comparisons between predictors in terms of influence on change during treatment. Importantly, these coefficients only allow comparisons across outcomes, when the latter are on the same scale. Hence, coefficients from regressions with SIAB-S scores at outcome can be compared with each other but not with the coefficients from the model with BMI as outcome. All analyses were performed with SPSS 25.

## Results

Of the 1238 patients treated between 2005 and 2012, 457 (36.9%) had missing data and 11 (0.9%) had implausible negative values of WE and were excluded from the analysis. The two groups did not differ with regard to SIAB-S total score, *t* (1041.2) = − 0.45, *p* = 0.653, LOS, *t* (1236) = 1.65, *p* = 0.099, and frequency of AN-type, χ^2^(1) = 1.71, *p* = 0.192. Patients with missing or implausible data had a lower admission BMI (*M* = 14.08, *SD* = 1.68) than included patients (*M* = 14.93, *SD* = 1.65), *t* (1236) = − 8.73, *p* <  0.001. Also, the excluded patients were younger (*M* = 25.91, *SD* = 8.69) than the included (*M* = 27.79, *SD* = 8.93), *t* (1236) = − 3.63, *p* <  0.001.

Of the included 770 female inpatients with AN, 305 (39.6%) had AN-R and 465 (60.4%) AN-BP. Their mean LOS was 91.22 days (*SD* = 43.11, range 21.00–292.00). The mean pre- to post-treatment changes were 2.54 (*SD* = 1.45, range − 2.08 – 7.90) for BMI, 0.74 (*SD* = 0.49, range − 2.08 – 2.51) for the SIAB-S total score, 1.09 (*SD* = 1.23, range − 2.62 – 4.00) for the bulimic symptoms scale, 1.21 (*SD* = 0.89, range − 2.55 – 3.55) for the slimness ideal scale, and 0.24 (*SD* = 0.28, range − 0.69 – 2.15) for the compensatory behaviors scale. Table 1 displays the descriptive statistics for the 24 weight history indices in the sample.

**Table 1:** Descriptive data of 24 weight history indices in 770 female inpatients with anorexia nervosa

Index	*M* (*SD*), range
Highest weight	59.52 (10.60), 38.00–130.00
Lowest weight	37.04 (5.93), 23.00–55.00
Highest BMI	21.30 (3.42), 14.66–43.44
Lowest BMI	13.24 (1.77), 8.66–17.30
Highest BMI percentage	1.01 (0.17), 0.68–2.06
Lowest BMI percentage	0.61 (0.08), 0.39–0.78
WS	17.84 (10.24), 1.00–89.00
WE	4.94 (3.96), 1.00–24.00
BMI suppression	6.37 (3.62), 0.14–29.74
BMI elevation	1.69 (1.40), 0.00–7.79
BMI suppression percentage	0.33 (0.18), 0.01–1.44
BMI elevation percentage	0.07 (0.06), 0.00–0.35
BMI range	8.06 (3.50), 1.88–30.07
PWC	0.29 (0.11), 0.02–0.68
Residual 1	0.00 (5.39), −15.42 – 14.22
Residual 2	0.00 (10.00), −17.23 – 70.47
Residual 3	0.00 (3.65), −7.31 – 17.72
Residual 4	0.00 (3.79), −17.05 – 6.76
h-AFW	76.20 (19.21), 40.00–210.60
l-AFW	40.37 (8.03), 23.00–70.00
Age at highest weight	18.72 (5.60), 10.00–59.00
Age at lowest weight	24.01 (8.27), 12–61
Years since highest weight	9.07 (7.56), 0.00–48.00
Years since lowest weight	3.79 (5.78), 0.00–45.00

*Note. BMI* body mass index, *WS* weight suppression, *WE* weight elevation, *PWC* percentage weight change, *Residual 1* highest on admission weight, *Residual 2* admission on highest weight, *Residual 3* lowest on admission weight, *Residual 4* admission on lowest weight, *h-AFW* highest age-factored weight, *l-AFW* lowest age-factored weight

Table 2 displays the bivariate correlation coefficients (Pearson’s *r*) for the indices. Most of the variables showed a range of correlations with other indices. Based on correlation coefficients ≥0.70, some expectable redundancy between the indices emerged. Some of the more “complex” indices showed very strong correlations with their simpler components, indicating that only little information is added in the transformation. For example, the correlation coefficients for the associations of WS with BMI suppression and BMI suppression percentage were 0.99 and 0.94, respectively. The correlation coefficient for the relationship of BMI suppression with BMI suppression percentage was 0.96. While the components of measures of WS correlated strongly with the more complex indices, their was no such pattern for measures of WE and their components. Residuals 1 (orthogonal to highest weight) and Residuals 2 (orthogonal to admission weight) had a correlation of 0.51 and 0.97 with WS, respectively. These results suggest that the variance in admission weight that is unexplained by highest weight, carries different information than WS. In turn, the information in highest weight that is unexplained by admission weight mostly overlaps with WS. Again, the pattern was different for Residuals 3 (orthogonal to lowest weight) and Residuals 4 (orthogonal to admission weight), which both showed a strong association with WE.

**Table 2:** Bivariate Pearson’s *r* correlation coefficients

	Highest weight	Lowest weight	Highest BMI	Lowest BMI	Highest BMI percentage	Lowest BMI percentage	WS	WE	BMI suppression	BMI elevation	BMI suppression percentage	BMI elevation percentage
Highest weight	1	0.39 ^***^	**0.91** ^***^	0.24 ^***^	**0.85** ^***^	0.20 ^***^	**0.85** ^***^	−0.11 ^**^	**0.80** ^***^	− 0.15 ^***^	**0.75** ^***^	− 0.12 ^**^
Lowest weight		1	0.17 ^***^	**0.88** ^***^	0.13 ^***^	**0.86** ^***^	−0.03	− 0.39	− 0.10 ^**^	− 0.44 ^***^	−0.12 ^**^	− 0.42 ^***^
Highest BMI			1	0.21 ^***^	**0.95** ^***^	0.18 ^***^	**0.88** ^***^	−0.12 ^**^	**0.89** ^***^	−0.13 ^***^	**0.85** ^***^	−0.10 ^**^
Lowest BMI				1	0.18 ^***^	**0.98** ^***^	−0.09 ^*^	− 0.46 ^***^	− 0.11 ^**^	− 0.48 ^***^	−0.11 ^**^	− 0.47 ^***^
Highest BMI percentage					1	0.16 ^***^	**0.85** ^***^	−0.11 ^**^	**0.86** ^***^	−0.12 ^**^	**0.91** ^***^	−0.11 ^**^
Lowest BMI percentage						1	−0.13 ^***^	− 0.41 ^***^	− 0.14 ^***^	− 0.42 ^***^	− 0.14 ^***^	− 0.47 ^***^
WS							1	−0.25 ^***^	**0.99** ^***^	−0.27 ^***^	0.94 ^***^	−0.23 ^***^
WE								1	−0.25^***^	**0.94** ^***^	−0.23 ^***^	**0.90** ^***^
BMI suppression									1	−0.27 ^***^	**0.96** ^***^	−0.24 ^***^
BMI elevation										1	−0.25 ^***^	**0.97** ^***^
BMI suppression percentage											1	− 0.23 ^***^
BMI elevation percentage												1
	BMI range	PWC	Residual 1	Residual 2	Residual 3	Residual 4	h-AFW	l-AFW	Age at highest weight	Age at lowest weight	Years since highest weight	Years since lowest weight
Highest weight	**0.77** ^***^	0.64 ^***^	0.00	**0.94** ^***^	0.05	0.21 ^***^	0.66 ^***^	0.18 ^***^	−0.02	0.07 ^*^	0.01	− 0.11 ^**^
Lowest weight	−0.28 ^***^	− 0.23 ^***^	0.68 ^***^	0.14 ^***^	0.00	0.64 ^***^	0.19 ^***^	**0.71** ^***^	− 0.01	−0.05	− 0.19 ^***^	− 0.18 ^***^
Highest BMI	**0.87** ^***^	**0.72** ^***^	−0.21 ^***^	0.03 ^***^	−0.05	− 0.15 ^***^	0.62 ^***^	0.02	−0.01	0.13 ^***^	0.08 ^*^	− 0.08 ^*^
Lowest BMI	−0.30 ^***^	−0.25 ^***^	0.55 ^***^	0.04	− 0.13 ^***^	0.66 ^***^	0.10 ^**^	0.64 ^***^	0.01	< 0.01	−0.15 ^***^	− 0.18 ^***^
Highest BMI percentage	**0.84** ^***^	**0.70** ^***^	−0.24 ^***^	**0.88** ^***^	−0.07	0.14 ^***^	**0.80** ^***^	0.05	−0.20 ^***^	0.02	0.10 ^**^	−0.09 ^*^
Lowest BMI percentage	−0.32 ^***^	− 0.30 ^***^	0.57 ^***^	< 0.01	− 0.08 ^*^	0.61 ^***^	0.10 ^**^	**0.75** ^***^	−0.02	− 0.10 ^**^	− 0.16 ^***^	− 0.09 ^*^
WS	0.91 ^***^	**0.92** ^***^	−0.51 ^***^	**0.97** ^***^	−0.28 ^***^	0.20 ^***^	0.61 ^***^	−0.18 ^***^	− 0.04	0.16 ^***^	0.08 ^*^	−0.15 ^***^
WE	0.12 ^**^	−0.33 ^***^	0.35 ^***^	−0.21 ^***^	**0.92** ^***^	**−0.96** ^***^	−0.09 ^*^	− 0.14 ^***^	0.04	0.14 ^***^	0.12 ^**^	0.39 ^***^
BMI suppression	**0.92** ^***^	**0.94** ^***^	−0.57 ^***^	**0.94** ^***^	−0.31 ^***^	0.17 ^***^	0.58 ^***^	−0.23 ^***^	−0.03	0.17 ^***^	0.10 ^**^	−0.14 ^***^
BMI elevation	0.12 ^**^	−0.35 ^***^	0.26 ^***^	−0.23 ^***^	0.83 ^***^	**−0.92** ^***^	−0.11 ^**^	− 0.17 ^***^	0.06	− 0.12 ^**^	0.13 ^***^	0.40 ^***^
BMI suppression percentage	**0.89** ^***^	**0.90** ^***^	−0.56 ^***^	**0.90** ^***^	−0.30 ^***^	0.16 ^***^	**0.75** ^***^	−0.18 ^***^	−0.22 ^***^	0.06	0.12 ^**^	−0.14 ^***^
BMI elevation percentage	0.14 ^***^	−0.30 ^***^	0.24 ^***^	−0.19 ^***^	**0.80** ^***^	**−0.89** ^***^	−0.12 ^**^	− 0.33 ^***^	0.09 ^*^	− 0.01	0.15 ^***^	0.28 ^***^
BMI range	1	0.83 ^***^	−0.49 ^***^	**0.88** ^***^	0.01	−0.19 ^***^	0.55 ^***^	−0.30 ^***^	−0.01	0.13 ^***^	0.16 ^***^	0.01
PWC		1	**−0.71** ^***^	**0.84** ^***^	−0.45 ^***^	0.20 ^***^	0.49 ^***^	−0.34 ^***^	−0.06	0.19 ^***^	0.12 ^**^	−0.17 ^***^
Residual 1			1	−0.33 ^***^	0.66 ^***^	−0.07 ^*^	−0.09 ^*^	0.62 ^***^	0.03	−0.19 ^***^	−0.13 ^***^	0.21 ^***^
Residual 2				1	−0.17 ^***^	0.22 ^***^	0.66 ^***^	−0.04	−0.03	0.13 ^***^	0.06	−0.14 ^***^
Residual 3					1	**−0.77** ^***^	−0.02	0.15 ^***^	0.04	−0.17 ^***^	0.04	0.34 ^***^
Residual 4						1	0.13 ^***^	0.34 ^***^	−0.03	0.10 ^**^	−0.16 ^***^	− 0.38 ^***^
h-AFW							1	0.20 ^***^	−0.56 ^***^	−0.24 ^***^	0.07	−0.12 ^**^
l-AFW								1	−0.14 ^***^	−0.42 ^***^	− 0.25 ^***^	0.14 ^***^
Age at highest weight									1	0.52 ^***^	−0.10 ^**^	0.10 ^**^
Age at lowest weight										1	0.54 ^***^	−0.23 ^***^
Years since highest weight											1	0.44 ^***^
Years since lowest weight												1

*Note*. ^*^Statistically significant at *p* = 0.05, ^**^at *p* = 0.01, and ^***^at *p* <  0.001. Coefficients ≥0.70 in boldface

*BMI* body mass index, *WS* weight suppression, *WE* weight elevation, *PWC* percentage weight change, *Residual 1* highest on admission weight, *Residual 2* admission on highest weight, *Residual 3* lowest on admission weight, *Residual 4* admission on lowest weight, *h-AFW* highest age-factored weight, *l-AFW* lowest age-factored weight

Also, the hypothesized orthogonality of the residuals 1 and 3 from highest and lowest weight, respectively, was confirmed by the respective correlation coefficients equaling 0.

Table 3 shows the rotated components matrix of the factor analysis. The rotation converged into five iterations. The first factor comprised indices pertaining to WS constructs as well as their components and explained 35.6% of the variance. The second factor included all indices that reflect WE but not its components (21.8% explained), confirming the dissociation between WE and lowest weight already observed in the correlation coefficients. The third factor included the components of WE indices as well as l-AFW and residual 1 (19.6% explained). The fourth factor included the ages at past weight (8.1% explained) and the fifth factor included the years since past weight (6.2% explained). Together, the five factors explain 91.3% of the variance.

**Table 3:** Results of the factor analysis. The indices are sorted according to their highest loading

Index	1	2	3	4	5
Residual 2	0.975				
Highest BMI	0.960				
WS	0.957				
BMI suppression	0.948				
Highest BMI percentage	0.947				
BMI suppression percentage	0.933				
BMI range	0.932				
Highest weight	0.920				
PWC	0.823				
h-AWF	0.713				
WE		0.970			
Residual 3		0.952			
BMI elevation		0.942			
BMI elevation percentage		0.917			
Lowest weight			0.930		
Lowest BMI percentage			0.914		
Lowest BMI			0.896		
l-AWF			0.815		
Residual 1			0.806		
Residual 4			0.415		
Age at highest weight				0.867	
Age at lowest weight				0.827	
Years since highest weight					0.949
Years since lowest weight					0.627

*Note*. *BMI* body mass index, *WS* weight suppression, *WE* weight elevation, *PWC* percentage weight change, *Residual 1* highest on admission weight, *Residual 2* admission on highest weight, *Residual 3* lowest on admission weight, *Residual 4* admission on lowest weight, *h-AFW* highest age-factored weight, *l-AFW* lowest age-factored weight

Table 4 shows the results of the multiple linear regression analyses with pre to post-treatment change in BMI as dependent variable. Almost all variables statistically significantly predicted change in BMI. The strongest predictors based on the absolute magnitude of standardized regression coefficients were Residual 1 and Residual 3, with higher values being associated with smaller gains in BMI, as well as PWC with higher values being associated with stronger BMI gains.

**Table 4:** Adjusted influence of weight history indices on BMI gains during inpatient treatment. Results of the multiple linear regression analyses

Predictor	Cor. *R*^2^	*B* (*SE*), 95% CI	*T*, *p*	*β*
Highest weight	0.265	0.004 (0.004), − 0.004; 0.013	1.01, 0.313	0.031
Lowest weight	0.276	−0.027 (0.008), − 0.042; − 0.013	− 3.62, < 0.001	− 0.112
Highest BMI	0.270	0.034 (0.013), 0.009; 0.060	2.62, 0.009	0.081
Lowest BMI	0.270	−0.065 (0.026), − 0.116; − 0.014	2.52, 0.012	−0.080
Highest BMI percentage	0.270	0.661 (0.262), 0.146; 1.176	2.52, 0.012	0.078
Lowest BMI percentage	0.272	−1.608 (0.569), −2.726; − 0.461	− 2.83, 0.005	− 0.090
WS	0.290	0.023 (0.004), 0.015; 0.032	5.31, < 0.001	0.164
WE	0.278	−0.044 (0.011), − 0.066; − 0.022	−3.93, < 0.001	− 0.121
BMI suppression	0.295	0.071 (0.012), 0.047; 0.095	5.78, < 0.001	0.178
BMI elevation	0.288	−0.159 (0.032), − 0.221; − 0.098	−5.05, < 0.001	−0.154
BMI suppression percentage	0.291	1.326 (0.247), 0.841; 1.810	5.37, < 0.001	0.165
BMI elevation percentage	0.286	−3.515 (0.723), −4.934; −2.095	− 4.86, < 0.001	−0.148
BMI oscillation	0.278	0.049 (0.013), 0.024; 0.074	3.85, < 0.001	0.119
PWC	**0.313**	**2.860 (0.387), 2.100; 3.619**	**7.39, < 0.001**	**0.227**
Residual 1	**0.315**	**−0.063 (0.008); − 0.079; − 0.046**	**−7.59, < 0.001**	**−0.233**
Residual 2	0.275	0.015 (0.004); 0.006, 0.024	3.36, 0.001	0.104
Residual 3	**0.296**	**−0.071 (0.012), − 0.095; − 0.048**	**−5.89, < 0.001**	**−0.180**
Residual 4	0.268	0.025 (0.012), 0.002; 0.048	2.13, 0.034	0.066
h-AFW	0.264	0.002 (0.002), −0.003; 0.006	0.74, 0.458	0.023
l-AFW	0.280	−0.023 (0.006), − 0.034; − 0.012	−4.09, < 0.001	−0.127
Age at highest weight	0.265	0.008 (0.008), −0.007; 0.024	1.03, 0.305	0.032
Age at lowest weight	0.265	0.006 (0.005), −0.005; 0.016	1.02, 0.309	0.031
Years since highest weight	0.265	−0.006 (0.006), − 0.018; 0.005	−1.06, 0.291	−0.033
Years since lowest weight	0.267	−0.014 (0.008), − 0.029; 0.001	− 1.85, 0.065	−0.057

*Note*. Regression coefficients are adjusted for length of stay. Corrected *R*^2^ = 0.265 for the regression model with only length of stay as predictor. Three best predictors based on *β* in boldface

*BMI* body mass index, *WS* weight suppression, *WE* weight elevation, *PWC* percentage weight change, *Residual 1* highest on admission weight, *Residual 2* admission on highest weight, *Residual 3* lowest on admission weight, *Residual 4* admission on lowest weight, *h-AFW* highest age-factored weight, *l-AFW* lowest age-factored weight

Table 5 shows the results of the multiple linear regression analyses with pre to post-treatment change in ED psychopathology (SIAB-S total score) as dependent variable. A range of variables including both highest and lowest weight showed positive associations with treatment changes. The strongest predictors were PWC, lowest BMI percentage, and lowest BMI with higher values being associated with more pronounced symptom reduction.

**Table 5:** Adjusted influence of weight history indices on changes in eating disorder psychopathology (SIAB-S total score) during inpatient treatment. Results of the multiple linear regression analyses

Predictor	Cor. *R*^2^	*B* (*SE*), 95% CI	*T*, *p*	*β*
Highest weight	0.047	0.005 (0.002), 0.002; 0.008	2.99, 0.003	0.107
Lowest weight	0.048	0.011 (0.004), 0.004; 0.018	3.12, 0.002	0.134
Highest BMI	0.045	0.014 (0.005), 0.004; 0.024	2.76, 0.006	0.098
Lowest BMI	**0.049**	**0.043 (0.013), 0.017; 0.069**	**3.29, 0.001**	**0.156**
Highest BMI percentage	0.048	0.324 (0.102), 0.123; 0.525	3.17, 0.002	0.112
Lowest BMI percentage	**0.049**	**0.973 (0.297), 0.389; 1.557**	**3.27, 0.001**	**0.160**
WS	0.046	0.005 (0.002), 0.002; 0.009	2.85, 0.004	0.107
WE	0.044	−0.012 (0.005), − 0.021; − 0.003	−2.55, 0.011	− 0.094
BMI suppression	0.045	0.014 (0.005), 0.004; 0.024	2.76, 0.006	0.104
BMI elevation	0.049	−0.043 (0.013), − 0.069; − 0.017	−3.29, 0.001	− 0.123
BMI suppression percentage	0.048	0.325 (0.102), 0.125; 0.526	3.18, 0.002	0.119
BMI elevation percentage	0.049	−0.990 (0.298), −1.575; − 0.404	−3.17, 0.001	−0.123
BMI oscillation	0.038	0.007 (0.005), −0.003; 0.018	1.46, 0.144	0.053
PWC	**0.049**	**0.592 (0.183), 0.233; 0.951**	**3.24, 0.001**	**0.183**
Residual 1	0.036	0.000 (0.006), −0.011; 0.011	−0.03, 0.979	− 0.002
Residual 2	0.046	0.005 (0.002), 0.002; 0.008	2.83, 0.005	0.101
Residual 3	0.040	−0.011 (0.006), − 0.022; 0.000	−1.90, 0.058	−0.081
Residual 4	0.046	0.013 (0.005), 0.004; 0.022	2.84, 0.004	0.100
h-AFW	0.047	0.003 (0.001), 0.001; 0.004	2.99, 0.003	0.105
l-AFW	0.042	0.006 (0.003), 0.001; 0.011	2.24, 0.025	0.094
Age at highest weight	0.043	−0.007 (0.003), −0.013; − 0.001	−2.34, 0.020	−0.083
Age at lowest weight	0.059	−0.009 (0.002), − 0.013; − 0.005	−4.33, < 0.001	−0.152
Years since highest weight	0.049	−0.007 (0.002), − 0.012; − 0.003	−3.25, 0.001	−0.114
Years since lowest weight	0.036	−0.001 (0.003), − 0.007; 0.005	−0.38, 0.702	− 0.014

*Note*. Regression coefficients are adjusted for length of stay and admission body mass index. Corrected *R*^2^ = 0.037 for the regression model with only length of stay and admission body mass index as predictors. Three best predictors based on *β* in boldface

*SIAB-S* Structured Inventory for Anorexic and Bulimic Disorders for DSM-IV and ICD-10 – Self-Rating, *Cor. R*^*2*^ corrected *R*^2^, *BMI* body mass index, *WS* weight suppression, *WE* weight elevation, *PWC* percentage weight change, *Residual 1* highest on admission weight, *Residual 2* admission on highest weight, *Residual 3* lowest on admission weight, *Residual 4* admission on lowest weight, h*-AFW* highest age-factored weight, *l-AFW* lowest age-factored weight

Table 6 shows the results of the multiple linear regression analyses with pre- to post-treatment change in bulimic symptoms (SIAB-S scale) as dependent variable. h-AFW, age at highest weight and age at lowest weight were the strongest predictors with higher values in the former being associated with larger treatment gains, and the latter two with smaller symptom reduction.

**Table 6:** Adjusted influence of weight history indices on changes in bulimic symptoms (SIAB-S scale) during inpatient treatment. Results of the multiple linear regression analyses

Predictor	Cor. *R*^2^	*B* (*SE*), 95% CI	*T*, *p*	*β*
Highest weight	0.058	0.009 (0.004), 0.001; 0.017	2.11, 0.035	0.075
Lowest weight	0.053	0.005 (0.009), − 0.012; 0.023	0.059, 0.558	0.025
Highest BMI	0.057	0.026 (0.013), 0.001; 0.051	2.05, 0.041	0.072
Lowest BMI	0.053	0.018 (0.033), −0.047; 0.082	0.53, 0.594	0.025
Highest BMI percentage	0.065	0.822 (0.254), 0.323; 1.321	3.23, 0.001	0.113
Lowest BMI percentage	0.053	0.471 (0.744), −0.989; 1.932	0.63, 0.526	0.031
WS	0.058	0.009 (0.004), 0.001; 0.018	2.12, 0.035	0.079
WE	0.052	0.004 (0.011), 0.019; 0.026	0.31, 0.759	0.011
BMI suppression	0.057	0.026 (0.013), 0.001; 0.051	2.05, 0.041	0.076
BMI elevation	0.053	−0.018 (0.033), − 0.082; 0.047	−0.53, 0.594	− 0.020
BMI suppression percentage	0.065	0.824 (0.254), 0.325; 1.323	3.24, 0.001	0.120
BMI elevation percentage	0.053	−0.491 (0.746), −1.955; 0.974	− 0.66, 0.511	−0.024
BMI oscillation	0.056	0.023 (0.13), −0.002; 0.048	1.83, 0.068	0.066
PWC	0.059	1.062 (0.456), 0.167; 1.956	2.33, 0.020	0.099
Residual 1	0.052	0.000 (0.014), −0.027; 0.027	0.02, 0.984	0.001
Residual 2	0.057	0.009 (0.004), 0.000; 0.017	1.99, 0.047	0.071
Residual 3	0.053	0.009 (0.014), 0.019; 0.037	0.63, 0.528	0.027
Residual 4	0.052	−0.001 (0.011), −0.024; 0.021	− 0.094, 0.925	−0.003
h-AFW	**0.077**	**0.010 (0.002), 0.006; 0.014**	**4.51, < 0.001**	**0.156**
l-AFW	0.054	0.007 (0.006), −0.006; 0.019	1.05, 0.295	0.044
Age at highest weight	**0.072**	**−0.031 (0.008), − 0.046; − 0.016**	**−3.99, < 0.001**	**−0.139**
Age at lowest weight	**0.074**	**−0.022 (0.005), − 0.032; − 0.012**	**−4.20, < 0.001**	**−0.147**
Years since highest weight	0.055	−0.009 (0.006), − 0.020; 0.002	−1.58, 0.114	− 0.056
Years since lowest weight	0.052	0.000 (0.008), −0.015; 0.015	0.02, 0.987	0.001

*Note*. Regression coefficients are adjusted for length of stay and admission body mass index. Corrected *R*^2^ = 0.054 for the regression model with only length of stay and admission body mass index as predictors. Three best predictors based on *β* in boldface

*SIAB-S* Structured Inventory for Anorexic and Bulimic Disorders for DSM-IV and ICD-10 – Self-Rating, *Cor. R*^*2*^ corrected *R*^2^, *BMI* body mass index, *WS* weight suppression, *WE* weight elevation, *PWC* percentage weight change, *Residual 1* highest on admission weight, *Residual 2* admission on highest weight, *Residual 3* lowest on admission weight, *Residual 4* admission on lowest weight, *h-AFW* highest age-factored weight, *l-AFW* lowest age-factored weight

Table 7 shows the results of the multiple linear regression analyses with pre to post-treatment change in slimness ideal (SIAB-S scale) as dependent variable. Lowest weight, lowest BMI, and lowest BMI percentage were the strongest predictor with higher values corresponding to better symptom reduction. Overall, indices including lowest weight displayed stronger associations with the outcome variable.

**Table 7:** Adjusted influence of weight history indices on changes in slimness ideal (SIAB-S scale) during inpatient treatment. Results of the multiple linear regression analyses

Predictor	Cor. *R*^2^	*B* (*SE*), 95% CI	*T*, *p*	*β*
Highest weight	0.014	0.008 (0.003), 0.002; 0.014	2.52, 0.012	0.091
Lowest weight	**0.043**	**0.035 (0.007), 0.023; 0.048**	**5.43, < 0.001**	**0.234**
Highest BMI	0.009	0.016 (0.009), − 0.003; 0.035	1.68, 0.093	0.061
Lowest BMI	**0.041**	**0.128 (0.024), 0.081; 0.175**	**5.33, < 0.001**	**0.253**
Highest BMI percentage	0.007	0.203 (0.190), −0.170; 0.576	1.07, 0.286	0.039
Lowest BMI percentage	**0.038**	**2.765 (0.544), 1.698; 3.832**	**5.09, < 0.001**	**0.249**
WS	0.011	0.007 (0.003), 0.000; 0.013	2.00, 0.046	0.076
WE	0.035	−0.041 (0.008), − 0.057; − 0.024;	−5.83, < 0.001	− 0.179
BMI suppression	0.009	0.016 (0.009), −0.003; 0.035	1.68, 0.093	0.064
BMI elevation	0.053^a^	−0.018 (0.033), − 0.082; 0.047	−0.53, 0.594	− 0.020
BMI suppression percentage	0.065	0.824 (0.254), 0.325; 1.323	3.24, 0.001	0.120
BMI elevation percentage	0.038	−2.784 (0.545), −3.854; − 1.713	−5.11, < 0.001	− 0.190
BMI oscillation	0.006	−0.003 (0.009), − 0.022; 0.015	−0.36, 0.719	− 0.013
PWC	0.014	0.865 (0.338), 0.201; 1.529	2.56, 0.011	0.111
Residual 1	0.007	0.010 (0.010), −0.010; 0.030	1.00, 0.316	0.061
Residual 2	0.012	0.007 (0.003), 0.001; 0.013	2.18, 0.030	0.079
Residual 3	0.053^a^	0.009 (0.014), −0.019; 0.037	0.63, 0.528	0.027
Residual 4	0.040	0.044 (0.008), 0.028; 0.060	5.27, < 0.001	0.186
h-AFW	0.006	0.000 (0.002), −0.003; 0.003	0.24, 0.981	0.001
l-AFW	0.018	0.014 (0.005), 0.005; 0.024	3.05, 0.002	0.129
Age at highest weight	0.006	0.003 (0.006), −0.008; 0.014	0.54, 0.590	0.019
Age at lowest weight	0.012	−0.009 (0.004), − 0.016; − 0.001	−2.52, 0.025	−0.081
Years since highest weight	0.029	−0.018 (0.004), − 0.026; − 0.010	−4.29, < 0.001	0.153
Years since lowest weight	0.010	− 0.010 (0.006), − 0.021; 0.001	−1.85, 0.065	−0.067

*Note*. Regression coefficients are adjusted for length of stay and admission body mass index. Corrected *R*^2^ = 0.007 for the regression model with only length of stay and admission body mass index as predictors. ^a^Increased *R*^2^ due to a suppression effect of BMI elevation on admission BMI, increasing the amount of variance explained by the latter. Three best predictors based on *β* in boldface

*SIAB-S* Structured Inventory for Anorexic and Bulimic Disorders for DSM-IV and ICD-10 – Self-Rating, *Cor. R*^*2*^ corrected *R*^2^, *BMI* body mass index, *WS* weight suppression, *WE* weight elevation, *PWC* percentage weight change, *Residual 1* highest on admission weight, *Residual 2* admission on highest weight, *Residual 3* lowest on admission weight, *Residual 4* admission on lowest weight, *h-AFW* highest age-factored weight, *l-AFW* lowest age-factored weight

Table 8 shows the results of the multiple linear regression analyses with pre to post-treatment change in compensatory behaviors (SIAB-S scale) as dependent variable. Lowest weight, lowest BMI, and lowest BMI percentage were the strongest predictors with higher values being associated with stronger treatment gains. Overall, indices including lowest weight were stronger predictors of the outcome variable.

**Table 8:** Adjusted influence of weight history indices on changes in compensatory behaviors (SIAB-S scale) during inpatient treatment. Results of the multiple linear regression analyses

Predictor	Cor. *R*^2^	*B* (*SE*), 95% CI	*T*, *p*	*β*
Highest weight	0.035	0.002 (0.001), 0.000; 0.004	2.05, 0.041	0.074
Lowest weight	**0.041**	**0.006 (0.002), 0.002; 0.010**	**3.06, 0.002**	**0.132**
Highest BMI	0.033	0.005 (0.003), − 0.001; 0.011	1.66, 0.097	0.059
Lowest BMI	**0.042**	**0.023 (0.007), 0.009; 0.038**	**3.13, 0.002**	**0.148**
Highest BMI percentage	0.032	0.079 (0.059), − 0.036; 0.194	1.34, 0.180	0.048
Lowest BMI percentage	**0.042**	**0.541 (0.170), 0.208; 0.874**	**3.19, 0.001**	**0.156**
WS	0.034	0.002 (0.001), 0.000; 0.004	1.79, 0.074	0.067
WE	0.036	−0.006 (0.003), − 0.011; − 0.001	−2.17, 0.030	− 0.081
BMI suppression	0.033	0.005 (0.003), − 0.001; 0.011	1.66, 0.097	0.063
BMI elevation	0.042	−0.023 (0.007), − 0.038; − 0.009	−3.13, 0.002	−0.117
BMI suppression percentage	0.032	0.079 (0.059), −0.036; 0.194	1.35, 0.177	0.051
BMI elevation percentage	0.043	−0.548 (0.170), − 0.882; − 0.214	−3.22, 0.001	−0.120
BMI oscillation	0.030	0.001 (0.003), −0.004; 0.007	0.44, 0.657	0.016
PWC	0.035	0.214 (0.105), 0.009; 0.419	2.04, 0.041	0.088
Residual 1	0.031	0.003 (0.003), −0.003; 0.009	0.87, 0.387	0.052
Residual 2	0.034	0.002 (0.001), 0.000; 0.004	1.77, 0.077	0.064
Residual 3	0.032	−0.005 (0.003), − 0.011; 0.002	−1.45, 0.146	−0.063
Residual 4	0.038	0.007 (0.003), 0.001; 0.012	2.53, 0.012	0.090
h-AFW	0.031	0.000 (0.001), −0.001; 0.001	0.78, 0.434	0.028
l-AFW	0.037	0.003 (0.001), 0.001; 0.006	2.38, 0.017	0.100
Age at highest weight	0.030	0.001 (0.002), −0.003; 0.004	0.40, 0.690	0.014
Age at lowest weight	0.034	−0.002 (0.001), − 0.005; 0.000	−1.77, 0.077	−0.063
Years since highest weight	0.040	−0.004 (0.001), − 0.006; − 0.001	−2.81, 0.005	−0.100
Years since lowest weight	0.031	−0.001 (0.002), − 0.005; 0.002	−0.78, 0.438	− 0.028

*Note*. Regression coefficients are adjusted for length of stay and admission body mass index. Corrected *R*^2^ = 0.031 for the regression model with only length of stay and admission body mass index as predictors. Three best predictors based on *β* in boldface

*SIAB-S* Structured Inventory for Anorexic and Bulimic Disorders for DSM-IV and ICD-10 – Self-Rating, *Cor. R*^*2*^ corrected *R*^2^, *BMI* body mass index, *WS* weight suppression, *WE* weight elevation, *PWC* percentage weight change, *Residual 1* highest on admission weight, *Residual 2* admission on highest weight, *Residual 3* lowest on admission weight, *Residual 4* admission on lowest weight, *h-AFW* highest age-factored weight, *l-AFW* lowest age-factored weight

**Fig. 1:**
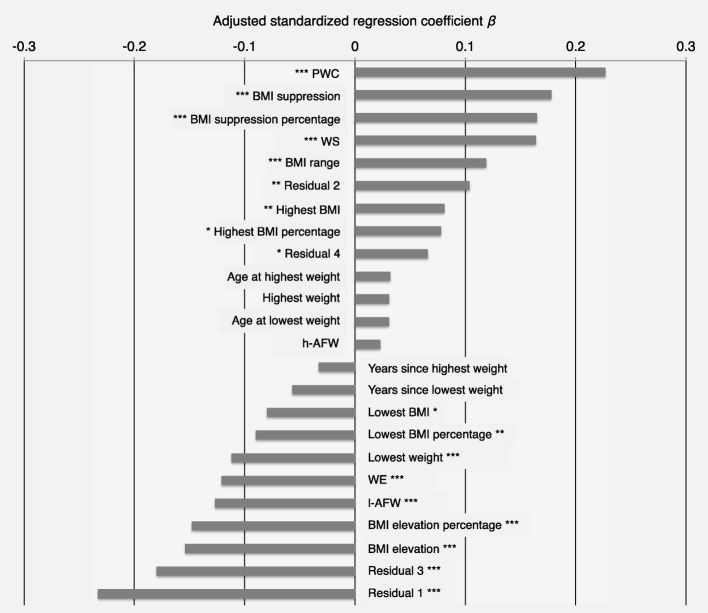
Adjusted standardized regression coefficients for the prediction of gain in body mass index during inpatient treatment for anorexia nervosa. *Note*. Regression coefficients adjusted for length of stay. * Statistically significant at *p* = 0.05, ** at *p* = 0.01, and *** at *p* < 0.001. BMI = body mass index, WS = weight suppression, WE = weight elevation, PWC = percentage weight change, Residual 1 = highest on admission weight, Residual 2 = admission on highest weight, Residual 3 = lowest on admission weight, Residual 4 = admission on lowest weight, h-AFW = highest age-factored weight, l-AFW = lowest age-factored weight

Table 9 summarizes the statistically significant association between all predictors and outcomes. Figure 1 displays the adjusted standardized regression coefficients of all indices from the regression models predicting BMI gain sorted according to their sign and magnitude.

**Table 9:** Summary of statistically significant associations between weight history indices and treatment changes

Index	BMI	Eating disorder psychopathology	Bulimic symptoms	Slimness ideal	Compensatory behaviors
Highest weight		+^**^	+^*^	+^*^	+^*^
Lowest weight	–^***^	+^**^		**+** ^***^	**+** ^**^
Highest BMI	+^**^	+^**^	+^*^		
Lowest BMI	–^*^	**+** ^**^		**+** ^***^	**+** ^**^
Highest BMI percentage	+^*^	+^**^	+^**^		
Lowest BMI percentage	–^**^	**+** ^**^		**+** ^***^	**+** ^**^
WS	+^***^	+^**^	+^*^	+^*^	
WE	–^***^	–^*^		–^***^	–^*^
BMI suppression	+^***^	+^**^	+^*^		
BMI elevation	–^***^	–^**^			–^**^
BMI suppression percentage	+^***^	+^**^	+^**^	+^**^	
BMI elevation percentage	–^***^	–^**^		–^***^	–^**^
BMI oscillation	+^***^				
PWC	**+** ^***^	**+** ^**^	+^*^	+^*^	–^*^
Residual 1	**–** ^***^				
Residual 2	+^**^	+^**^	+^*^	+^*^	
Residual 3	**–** ^***^				
Residual 4	+^*^	+^*^		+^***^	+^*^
h-AFW		+^**^	**+** ^***^		
l-AFW	–^***^	+^*^		+^**^	+^*^
Age at highest weight		–^*^	**–** ^***^		
Age at lowest weight		–^***^	**–** ^***^	–^*^	
Years since highest weight		–^**^		–^***^	–^**^

*Note*. No statistically significant association with “years since lowest weight”. The signs of the three best predictors based on *β* for each outcome in boldface

*BMI* body mass index, *WS* weight suppression, *WE* weight elevation, *PWC* percentage weight change, *Residual 1* highest on admission weight, *Residual 2* admission on highest weight, *Residual 3* lowest on admission weight, *Residual 4* admission on lowest weight, *h-AFW* highest age-factored weight, *l-AFW* lowest age-factored weight

^*^Statistically significant at *p* = 0.05, ^**^ at *p* = 0.01, and ^***^ at *p* < 0.001. + = higher index values associated with more pronounced improvement, − = higher index values associated with less pronounced improvement

## Discussion

Our study explored a wide range of indices that measure weight history. We investigated the (1) components and different measures of WS [2], (2) examined developmental aspects of weight history [3], (3) applied WE to a clinical population, and (4) introduced new indices based on the age at past weight. Bearing in mind the limitations that come with an exploratory study like ours, the investigation of redundancy and underlying constructs suggested five groups of weight history indices that can be summarized as “highest weight and WS”, “WE”, “lowest weight”, “age at past weight”, and “years since past weight”. Below, we will outline the respective findings for these groups and suggest future lines of research. Overall, our findings need replication in independent samples, in-depth comparisons within groups of indices (e.g. all related to WS), transference to long-term follow-up, and linkage to behavioral and metabolic aspects of EDs.

### Highest weight and WS

WS is the most commonly examined weight history variable [10]. Yet, simple difference score based operationalizations were criticized for not taking into account developmental aspects and its individual components are understudied [2]. A proposed solution was to relate highest weight or the difference score to what would be expected at the given age. Correlational and factor analyses suggested a great deal of redundancy in the indices related to WS and their components. All indices of WS, including BMI suppression percentage and PWC as more complex and development-sensitive indices showed strong correlations with each other as well as with simple scores like highest weight and highest BMI. WS and BMI suppression had a correlation coefficient of 0.99, suggesting almost complete congruency between the distributions of the two variables. Factor analysis supported these observations with yielding a common factor for measures of WS and highest weight. These results suggest that measures of WS are highly dependent on highest weight and most of the contained information in WS seems to be traceable to highest weight, which is in line with other studies [10]. While Residuals 1, which are orthogonal to highest weight, were moderately or weakly related to respective indices, Residuals 2, which are orthogonal to admission weight, were strongly associated with measures of highest weight and WS. Accordingly, Residuals 2 loaded the strongest on factor 1, followed by highest BMI and WS, while Residuals 1 loaded on the same factor as components of WE. Indices that account for developmental aspects did not load stronger on the factor than simpler variables. These findings are paralleled by the regression analyses, in which no marked differences in predictive power emerged. Measures of highest weight and WS were among the strongest predictors for change in BMI and general ED psychopathology, but not for the other outcomes. The importance of highest weight is further emphasized by the fact that BMI range, which combines highest and lowest weight, also had its highest loading on factor 1. In sum, these results confirm the simple kilogram-based operationalization of WS, highlight the dominance of highest weight in measures of WS, and propose regression residuals orthogonal to admission weight as subject of future investigations [10].

### We

WE, the difference between current and past lowest weight, is a fairly neglected construct in the context of weight history [11]. Surprisingly, measures of lowest weight and WE did not load on the same factor and showed only small to moderate correlations. Rather, the dissociation between WE and lowest weight as well as the high factor loading of Residuals 3, which contain the variance in admission weight that is independent of lowest weight, suggest that admission weight might be the dominant variable in measures of WE. Yet, this relationship is still ambiguous, as WE showed high correlations with both Residuals 3 and 4, with the latter representing the variance in lowest weight unexplained by admission weight. Parallel to measures of WS, development-sensitive indices showed lower factor loadings than the kilogram-based measure and regression residuals orthogonal to lowest weight (Residuals 3) and were not stronger predictors in the regression analyses. Measures of WE were among the strongest predictors for changes in slimness ideal and inappropriate compensatory behaviors with lower values relating to more improvement. This association is novel and opens interesting lines of research. Future studies might also investigate the dependence of kilogram-based WE on admission weight and Residuals 3 as potentially useful operationalization of WE. Also, future investigations might build on data from community samples [11] and our regression analyses, which suggest an influence of measures of WE on treatment outcome in AN, and transfer the construct to a clinical setting.

### Lowest weight

The third factor summarized the different variables measuring lowest weight. The kilogram-based index had the highest loading, followed by the development-sensitive lowest BMI percentage. As a measure of variance in lowest weight unexplained by admission weight, Residuals 4 fit into this group of indices, though its loading was rather weak. Yet surprising is the inclusion of Residuals 1 (variance in admission weight unexplained by highest weight), as we would rather expect this index to load on the factor of WE due to its emphasis on admission weight. The correlation matrix suggests moderate positive associations of Residuals 1 with lowest weight, lowest BMI, and lowest BMI percentage and a strong negative association with PWC. This ambiguity needs to be investigated by future studies. Even more than WE, measures of lowest weight predicted changes in slimness ideal and compensatory behaviors. Higher values (i.e. less extreme) related to better improvement, rendering lowest weight an important, though previously overlooked, predictor for the amenability of core ED symptoms to intense and specialized treatment. This finding might inform the refinement and tailoring of treatment for AN.

### Age at past weight and years since past weight

The two last factors represent the four variables that are exclusively age-based, with age at past weight and years since past weight loading on separate factors. h-AFW, which combines highest weight with age at highest weight, and its counterpart l-AFW loaded on factors 1 and 3, respectively. Together, these findings suggest that weight dominates h-AFW and l-AFW and the information contained in the age variables might rather be obscured by the combination with weight. Since the age variables only showed the expected moderate correlations with h-AFW and l-AFW, it seems that they carry unique information about weight history. In the regression analyses, the age variables were especially predictive of change in ED pathology, bulimic symptoms, and slimness ideal. Higher age at both lowest and highest weight since puberty were especially associated with less improvement in bulimic symptoms. More years since lowest weight further predicted less improvement in slimness ideal. While the here introduced variables of AFW did not seem to be necessary additions to the study of age history, our findings confirm Lowes’ case for examining age in the context of weight history [2] and warrant their detailed investigation in future studies.

### Strength and limitations

Strength of our study include the large sample size and the naturalistic setting. Among the limitations are the self-report measurement of weight history, which directly concerns the reliability and validity of our findings. However, a study examining the accuracy of self-report values for past weight found only a small mean error after a 20-year period [18]. Also, as is common in routine care, we had a fairly large proportion of missing data, which may have introduced bias to our study and its findings. The lack of an independent sample to validate our results precludes any statements whose validity reaches beyond your sample.

## Conclusion

Twenty-one of the 24 examined indices predicted response to inpatient treatment for AN, among those many previously neglected scores. Different dimensions of weight history emerged, within which the indices showed high redundancy. Importantly, these dimensions predicted change in different aspects of AN, suggesting specific associations between facets of past weight and current weight-related, cognitive, behavioral, and emotional symptoms of AN. Given their high correlations, indices from the same dimensions are likely to make similar predictions with regard to treatment outcome. Weight history is, hence, an important source of knowledge in the investigation and treatment of AN and needs to be examined more closely in future studies.

## Abbreviations

AFW: Age factored weight
AN: Anorexia nervosa
AN-BP: Binge/purge type of anorexia nervosa
AN-R: Restrictive type of anorexia nervosa
BMI: Body mass index
BN: Bulimia nervosa
ED: Eating disorders
h-AFW: Highest age-factored weight
l-AFW: Lowest age-factored weight
LOS: Length of stay
PWC: Percentage weight change
SIAB-S: Structured Inventory for Anorexic and Bulimic Disorders for DSM-IV and ICD-10 – Self-Rating
WE: Weight elevation
WS: Weight suppression

## References

[1] Lowe MR, Brownell KD, Walsh BT (2018). Weight suppression. Eat Disord Obes a Compr Handb.

[2] Lowe MR, Piers AD, Benson L. Weight suppression in eating disorders: a research and conceptual update. Curr Psychiatry Rep. 2018;20:1–12. 10.1007/s11920-018-0955-2 30155651

[3] Gorrell S, Reilly EE, Schaumberg K, Anderson LM, Donahue JM (2018). Weight suppression and its relation to eating disorder and weight outcomes: a narrative review. Eat Disord.

[4] Hessler JB, Diedrich A, Greetfeld M, Schlegl S, Schwartz C, Voderholzer U (2018). Weight suppression but not symptom improvement predicts weight gain during inpatient treatment for bulimia nervosa. Eur Eat Disord Rev.

[5] Berner LA, Shaw JA, Witt AA, Lowe MR (2013). The relation of weight suppression and body mass index to symptomatology and treatment response in anorexia nervosa. J Abnorm Psychol.

[6] Carter FA, Boden JM, Jordan J, McIntosh VVW, Bulik CM, Joyce PR (2015). Weight suppression predicts total weight gain and rate of weight gain in outpatients with anorexia nervosa. Int J Eat Disord..

[7] Bodell LP, Racine SE, Wildes JE (2016). Examining weight suppression as a predictor of eating disorder symptom trajectories in anorexia nervosa. Int J Eat Disord..

[8] Witt AA, Berkowitz SA, Gillberg C, Lowe MR, Råstam M, Wentz E (2014). Weight suppression and body mass index interact to predict long-term weight outcomes in adolescent-onset anorexia nervosa. J Consult Clin Psychol.

[9] Butryn ML, Juarascio A, Lowe MR (2011). The relation of weight suppression and BMI to bulimic symptoms. Int J Eat Disord..

[10] Schaumberg K, Anderson LM, Reilly EE, Gorrell S, Anderson DA, Earleywine M (2016). Considering alternative calculations of weight suppression. Eat Behav.

[11] Goodman Erica L., Baker Jessica H., Peat Christine M., Yilmaz Zeynep, Bulik Cynthia M., Watson Hunna J. (2018). Weight suppression and weight elevation are associated with eating disorder symptomatology in women age 50 and older: Results of the gender and body image study. International Journal of Eating Disorders.

[12] Stice E, Shaw HE (2002). Role of body dissatisfaction in the onset and maintenance of eating pathology: a synthesis of research findings. J Psycosomatic Res.

[13] Fichter M, Quadflieg N (2000). Comparing self- and expert rating: a self-report screening version (SIAB-S) of the structured interview for anorexic and bulimic syndromes for DSM-IV and ICD-10 (SIAB-EX). Eur Arch Psychiatry Clin Neurosci.

[14] Fichter M, Quadflieg N (2001). The structured interview for anorexic and bulimic disorders for DSM-IV and ICD-10 (SIAB-EX): reliability and validity. Eur Psychiatry.

[15] Kromeyer-Hauschild K, Wabitsch M, Kunze D, Geller F, Geiß HC, Hesse V (2001). Percentiles of body mass index in children and adolescents evaluated from different regional German studies. Monatsschr Kinderheilkd.

[16] Hemmelmann C, Brose S, Vens M, Hebebrand J, Ziegler A (2010). Percentiles of body mass index of 18-80-year-old German adults based on data from the second National Nutrition Survey. Dtsch Med Wochenschr.

[17] Le LKD, Barendregt JJ, Hay P, Mihalopoulos C (2017). Prevention of eating disorders: a systematic review and meta-analysis. Clin Psychol Rev.

[18] Tamakoshi K, Yatsuya H, Kondo T, Hirano T, Hori Y, Yoshida T (2003). The accuracy of long-term recall of past body weight in Japanese adult men. Int J Obes.

