# Correction to: Dimensions within 24 weight history indices and their association with inpatient treatment outcome in adults with anorexia nervosa: analysis of routine data

**DOI:** 10.1186/s40337-020-0283-x

**Published:** 2020-02-05

**Authors:** Johannes Baltasar Hessler, Sandra Schlegl, Martin Greetfeld, Ulrich Voderholzer

**Affiliations:** 1 Schoen Clinic Roseneck, Prien, Germany; 2 Department of Psychiatry and Psychotherapy, University Hospital of Munich (LMU), Munich, Germany; 3 Department of Psychiatry and Psychotherapy, University Hospital of Freiburg, Freiburg, Germany

**Correction to: J Eat Disord**

**https://doi.org/10.1186/s40337-019-0249-z**

In the publication of this article [1], there is an error with an abbreviation.

The last sentence of the results paragraph in the abstract reads as follows: “While measures of WE related more to weight gain and general ED Psychopathology, indices including lowest weight were stronger predictors of changes in slimness ideal and inappropriate compensatory behaviors.” Instead of WE (weight elevation), however, the abbreviation should say WS (weight suppression). This has now been included in this correction article.

## References

[1] Hessler JB, Schlegl S, Greetfeld M (2019). Dimensions within 24 weight history indices and their association with inpatient treatment outcome in adults with anorexia nervosa: analysis of routine data. J Eat Disord.

